# Supplementary material for: Maternal mid-pregnancy dietary patterns and inflammatory bowel disease in offspring from a prospective cohort study
Source: Commun Med (Lond). 2025 Dec 29;6:80. doi: 10.1038/s43856-025-01338-z (PMC12873246; doi:10.1038/s43856-025-01338-z)
Supplement: Supplementary file 2 — Supplementary Material [file 43856_2025_1338_MOESM2_ESM.pdf]

Supplementary material

Maternal mid-pregnancy dietary patterns and inflammatory bowel disease in offspring from a prospective cohort study

Olivia Mariella Anneberg, Sjurdur Frodi Olsen, Anne Vinkel Hansen, Mette Julsgaard, Anne Ahrendt Bjerregaard, Thorhallur Ingi Halldorsson, Tine Jess, & Maiara Brusco De Freitas

Table of contents

Supplementary Figure 1. Kaplan-Meier curves of the cumulative incidence for pediatric-onset (≤18 years old) inflammatory bowel disease in offspring from the Danish National Birth Cohort. Curves are presented separately for each maternal dietary pattern.. 1

Supplementary Table 1. Food groups used for k-means cluster analysis.* 2

Supplementary Table 2. Characteristics of the included vs excluded mother-child pairs. 3

Supplementary Table 3. K-means cluster centroids for all analyzed food groups across the identified clusters.* 4

Supplementary Table 4. Maternal dietary patterns during pregnancy and risk of pediatric-onset inflammatory bowel disease in the offspring after further adjusting for offspring antibiotics use during the first life year.. 5

Supplementary Figure 1. Kaplan-Meier curves of the cumulative incidence for pediatric-onset (≤18 years old) inflammatory bowel disease in offspring from the Danish National Birth Cohort. Curves are presented separately for each maternal dietary pattern.

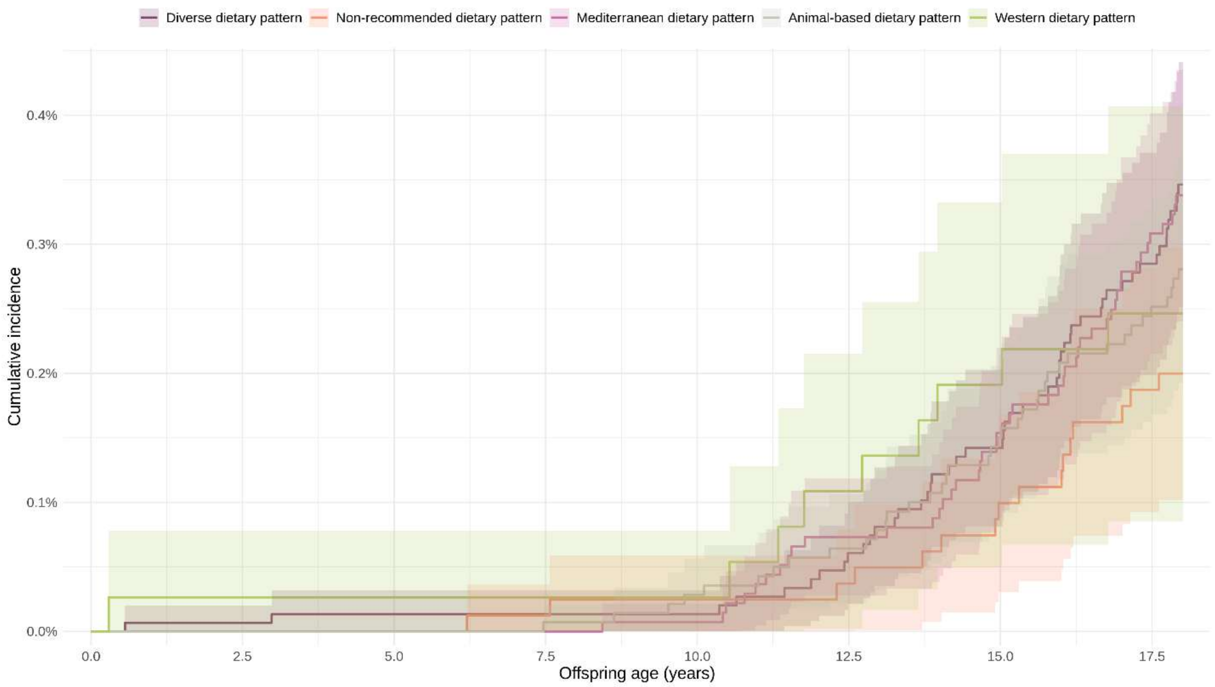

Supplementary Table 1. Food groups used for k-means cluster analysis.*

Food group	Foods
Fermented dairy	Buttermilk; Yoghurt naturel
Unfermented dairy	Milk 0.1% fat; Milk 0.5% fat; Milk 1.5% fat; Milk 3.5% fat
Sweetened dairy	Chocolate milk; Yoghurt with fruit
Cheese	Blue cheese; Brie and camembert; Cottage cheese; Cream cheese and cheese spread; Sliced cheese
Whole grain products	Crisp bread; Müsli; Oats and oatmeal; Rye bread; Wheat bread
Refined grain products	Breakfast cereals (Cornflakes, Frosties, etc.); Pasta; Rice and bulgur; White bread
Red meat	Beef steak; Casserole with beef; Casserole with chopped meat; Casserole with lamb; Casserole with pork; Fried ribs; Ham and smoked saddle of pork; Ham schnitzel; Hamburgers and meat balls; Lamb chop; Lasagna; Meat loaf; Pork chop; Rissoles; Roast beef and roast veal; Roast lamb; Roast pork; Tenderloin steak; Veal chop; Veal schnitzel
Processed meat	Chicken and turkey (toppings); Cod roe; Fillet of pork and salted veal; Frankfurters; Ham and smoked saddle of pork; Liver paste; Mayonnaise toppings with fish; Mayonnaise toppings with meat; Meat sausage; Pork sausage; Roast beef (topping); Roast pork (toppings); Salami
Offal	Casserole with heart; Casserole with liver; Fried liver
Poultry	Casserole with chicken; Casserole with turkey; Chicken; Duck or goose; Pheasant; Turkey
Eggs	Eggs; Omelet; Soufflé
Oily fish	Halibut; Herring; Mackerel; Mackerel in tomato sauce; Marinated herring; Salmon; Sardines canned in oil; Smoked herring and mackerel; Smoked salmon and halibut; Trout
Lean fish	Canned tuna; Cod or coalfish; Fish cake; Fried fillet of fish; Garpike; Plaice or flounder; Seal or whale
Shellfish	Caviar; Shellfish; Shrimp
Fats	Butter or spread on bread; Fats used for frying; Fats used for sauces; Fats used for casseroles; Melted butter and margarine
Vegetables	Bell pepper; Broccoli; Brussels sprouts; Carrots; Cauliflower; Corn; Cucumber; Eggplant; Garlic; Kale; Leak; Lettuce and iceberg; Mushroom; Onion; Other mushrooms; Red cabbage; Spinach; Tomato; Vegetable dishes; Vegetable mix; Vegetarian pie; Vegetarian beef; White cabbage; Zucchini
Legumes	Green beans; Peas
Fruits	Apple; Avocado; Banana; Dried apricots; Dried prunes; Dried raisins; Dried figs; Dried dates; Fig topping; Fruit salad; Grapefruit; Grapes; Kiwi; Oranges and mandarins; Peach and nectarine; Pear; Plum; Strawberries; Watermelon; Other melon
Nuts	Almonds and other nuts; Peanuts and pistachio nuts
Soy products	Soy milk; Soybeans (tofu or miso)
Potatoes	Baked potatoes; Boiled potatoes; Creamed potatoes; French fries; Fried potatoes; Mashed potatoes; Potato salad
Juices	Apple juice; Carrot juice; Grape juice; Orange juice; Tomato juice; Orange juice with calcium
Dressings and sauces	Bearnaise or hollandaise sauce; Dressings for salads; Mayonnaise; Remoulade; Sauce with fat; Sauce without fat
Soups	Beef soup; Chicken soup; Fish soup; Tomato soup; Vegetable soup
Snacks	Chips; Cracklings; Popcorn
Candy and chocolate	Chocolate; Chocolate topping; Chocolate spread; Drops; Liquorice; Liquorice allsorts; Mixed candy; Toffees; Wine gum
Desserts	Buttermilk koldskål; Cookies or biscuits; Cream cake; Danish pastry; Fruit pie; Fruit porridge; Ice cream; Ice lolly; Pancakes and æbleskiver; Sponge cake
Honey and jam	Honey; Jam
Coffee and tea	Coffee; Tea
Beer	Light beer; Regular beer; Strong beer
Wine	Port wine; Red wine; Rosé wine; White wine
Liquor	Liquor
Water	Regular water; Sparkling water
Soft drinks with sugar	Fruit syrup and water with sugar; Soda pop with sugar
Soft drinks sugar-free	Fruit syrup and water without sugar ("diet"); Soda pop without sugar ("diet")
Pizza and burger	Burger; Pizza
Sushi	Sushi

*The table presents the 37 food groups used for dietary pattern analysis, as well as the different food items that belonged to each group.

Supplementary Table 2. Characteristics of the included vs excluded mother-child pairs.

	Total (n=96817)	Excluded (n=40720)	Included (n=56097)	p-value
Maternal age at birth, years				<0.001
Median (IQR)	30.0 (27.0-33.0)	30.0 (27.0-33.0)	30.0 (27.0-33.0)	
Missing data, n (%)	0 (0.0%)	0 (0.0%)	0 (0.0%)	
Maternal educational level				<0.001
Primary, n (%)	11674 (12.1%)	4854 (11.9%)	6820 (12.2%)	
Secondary or post-secondary, n (%)	35805 (37.0%)	13784 (33.9%)	22021 (39.3%)	
Tertiary, n (%)	42099 (43.5%)	14843 (36.5%)	27256 (48.6%)	
Missing data, n (%)	7239 (7.5%)	7239 (17.8%)	0 (0.0%)	
Maternal pre-pregnancy BMI, kg/m^2				0.114
Median (IQR)	22.6 (20.7-25.4)	22.7 (20.7-25.6)	22.6 (20.7-25.4)	
Missing data, n (%)	7591 (7.8%)	7591 (18.6%)	0 (0.0%)	
Maternal alcohol intake in 2nd trimester				<0.001
Yes, n (%)	43276 (44.7%)	14852 (36.5%)	28424 (50.7%)	
No, n (%)	44538 (46.0%)	16865 (41.4%)	27673 (49.3%)	
Missing data, n (%)	9003 (9.3%)	9003 (22.1%)	0 (0.0%)	
Maternal smoking in 2nd trimester				<0.001
Yes, n (%)	15933 (16.5%)	7018 (17.2%)	8915 (15.9%)	
No, n (%)	71858 (74.2%)	24676 (60.6%)	47182 (84.1%)	
Missing data, n (%)	9026 (9.3%)	9026 (22.2%)	0 (0.0%)	
Maternal diet quality in 2nd trimester				<0.001
Median (IQR)	22.3 (17.9-27.3)	22.8 (18.1-27.9)	22.2 (17.8- 27.1)	
Missing data, n (%)	23011 (23.8%)	23011 (56.5%)	0 (0.0%)	
Maternal nutritional supplement use in 2nd trimester				<0.001
Yes, n (%)	85751 (88.6%)	30832 (75.7%)	54919 (97.9%)	
No, n (%)	3026 (3.1%)	1848 (4.5%)	1178 (2.1%)	
Missing data, n (%)	8040 (8.3%)	8040 (19.7%)	0 (0.0%)	
Maternal energy intake in 2nd trimester, kJ/day				<0.001
Median (IQR)	9810 (8229-11609)	9710 (8035-11584)	9830 (8277-11616)	
Missing data, n (%)	25462 (26.3%)	25462 (62.5%)	0 (0%)	
Maternal antibiotics use in pregnancy				<0.001
0 courses, n (%)	66861 (69.1%)	27545 (67.6%)	39316 (70.1%)	
1-2 courses, n (%)	22819 (23.6%)	9727 (23.9%)	13092 (23.3%)	
≥3 courses, n (%)	7137 (7.4%)	3448 (8.5%)	3689 (6.6%)	
0 courses, n (%)	0 (0.0%)	0 (0.0%)	0 (0.0%)	
Parental IBD diagnosis				0.105
Yes, n (%)	1081 (1.1%)	428 (1.1%)	653 (1.2%)	
No, n (%)	95736 (98.9%)	40292 (98.9%)	55444 (98.8%)	
Missing data, n (%)	0 (0.0%)	0 (0.0%)	0 (0.0%)	
Child's sex				0.649
Girl, n (%)	47064 (48.6%)	19780 (48.6%)	27284 (48.6%)	
Boy, n (%)	49576 (51.2%)	20763 (51.0%)	28813 (51.4%)	
Preterm delivery				<0.001
Yes (<37 weeks), n (%)	6201 (6.4%)	4175 (10.3%)	2026 (3.6%)	
No (≥37 weeks), n (%)	88945 (91.9%)	35590 (87.4%)	53355 (95.1%)	
Missing data, n (%)	1671 (1.7%)	955 (2.3%)	716 (1.3%)	
Child's antibiotics use in first year of life				<0.001
Yes, n (%)	39648 (41.0%)	16440 (40.4%)	23208 (41.4%)	
No, n (%)	57169 (59.0%)	24280 (59.6%)	32889 (58.6%)	
Missing data, n (%)	0 (0.0%)	0 (0.0%)	0 (0.0%)	
Any breastfeeding duration[1], days				<0.001
Median (IQR)	180 (134-180)	180 (120-180)	180 (134-180)	
Missing data, n (%)	26614 (27.5%)	15980 (39.2%)	10634 (19.0%)	
Exclusive breastfeeding duration[1], days				<0.001
Median (IQR)	120 (90-150)	120 (90-150)	120 (90-150)	
Missing data, n (%)	44519 (46.0%)	23147 (56.8%)	21372 (38.1%)	
Mode of delivery				<0.001
Vaginal, n (%)	80181 (82.8%)	32354 (79.5%)	47827 (85.3%)	
Cesarean, n (%)	16257 (16.8%)	8005 (19.7%)	8252 (14.7%)	
Missing, n (%)	379 (0.4%)	361 (0.9%)	18 (<0.1%)	
Disease subtype				0.421
No disease, n (%)	96556 (99.7%)	40620 (99.8%)	55936 (99.7%)	
Crohn's disease, n (%)	144 (0.1%)	55 (0.1%)	89 (0.2%)	
Ulcerative colitis, n (%)	117 (0.1%)	45 (0.1%)	72 (0.1%)	

*Data are expressed as median (IQR) for continuous variables and counts (%) for categorical variables, which were compared between groups using two-sided Mann-Whitney U and X^2 tests, respectively. [1]Truncated at 180 days. Abbreviations: BMI, body mass index. IBD, inflammatory bowel disease.

Supplementary Table 3. K-means cluster centroids for all analyzed food groups across the identified clusters.*

	Cluster 1 (Diverse dietary pattern)	Cluster 2 (Non-recommended dietary pattern)	Cluster 3 (Mediterranean dietary pattern)	Cluster 4 (Animal-based dietary pattern)	Cluster 5 (Western dietary pattern)
Fermented dairy	0.3754813	-0.35140711	0.391211737	-0.022969843	-0.255418897
Unfermented dairy	0.2983743	-0.15125452	-0.055614442	-0.104377500	0.056089032
Sweetened dairy	0.2964748	-0.16409845	-0.194098861	-0.101105637	0.203224560
Cheese	0.5206183	-0.50268032	0.335803486	0.180529373	-0.192027999
Whole grain products	0.5114623	-0.57687093	0.447536578	0.154095003	-0.219738794
Refined grain products	0.2682446	-0.18185501	-0.296289543	-0.087379313	0.331597335
Red meat	0.7652613	-0.36764963	-0.463538872	-0.222625539	0.426101920
Processed meat	0.6461977	-0.47164888	-0.385738781	0.085045999	0.436098609
Offal	0.3100997	-0.19544811	-0.026024769	0.152110119	-0.002651577
Poultry	0.6473161	-0.45256116	0.213070049	-0.227406445	-0.086820991
Eggs	0.6082214	-0.44415033	0.006919942	0.126997798	0.038424863
Oily fish	0.9183761	-0.62492095	0.239690713	-0.007647809	-0.157712174
Lean fish	0.9242904	-0.63255977	0.165584916	0.131171111	-0.116700484
Shellfish	0.7468770	-0.43356917	0.091077341	-0.179162383	-0.053258905
Fats	0.3196836	-0.27551301	-0.257174252	0.014929962	0.326657678
Vegetables	1.0637079	-0.54413290	0.406120532	-1.682128867	-0.053614202
Legumes	0.9110831	-0.43574538	0.151056828	-0.643164200	-0.083835633
Fruits	0.9013769	-0.57041290	0.477804080	-0.843307208	-0.220305444
Nuts	0.7110988	-0.46194404	0.210313118	-0.066723214	-0.151360892
Soy products	0.2623721	0.05726960	0.332701677	-2.444703743	0.093424305
Potatoes	0.7305207	-0.43307521	-0.419545147	-0.005659502	0.408982114
Juices	0.5453242	-0.35340713	0.109204991	-0.511454143	0.050009899
Dressings and sauces	0.7027734	-0.30631940	-0.030644007	-2.023807302	0.436314915
Soups	0.6592877	-0.10046609	0.242507288	-2.462968418	0.112755819
Snacks	0.4930783	-0.34970794	-0.381644526	-0.019677427	0.429629847
Candy and chocolate	0.4734043	-0.26936740	-0.366107444	-0.574520931	0.490319645
Desserts	0.8194788	-0.50741412	-0.113691880	-0.814569992	0.335231498
Honey and jam	0.4486732	-0.19341593	0.267116787	-1.738845025	0.110034690
Coffee and tea	0.3160421	-0.35055901	0.252987288	0.191308857	-0.142600925
Beer	0.3754486	-0.27646585	0.036278853	0.006923562	0.012723227
Wine	0.4961486	-0.42079957	0.150492424	-0.037709103	-0.019405931
Liquor	0.1764231	-0.12604953	-0.056124378	0.022816176	0.068707602
Water	0.4279795	-0.38720707	0.446041463	-0.117267294	-0.280740594
Soft drinks with sugar	0.2106370	-0.21714967	-0.251067520	0.105174056	0.303979100
Soft drinks sugar-free	0.1070400	0.01457122	-0.216912768	-0.020904432	0.143447784
Pizza and burger	0.3772863	-0.21958268	-0.385291085	-0.081142153	0.391385737
Sushi	0.1903623	-0.01315953	0.083065975	-0.642794737	-0.012218243

*The presented values refer to the mean (z-score normalized) intake of each food group across the five diet clusters, as illustrated in Figure 2.

Supplementary Table 4. Maternal dietary patterns during pregnancy and risk of pediatric-onset inflammatory bowel disease in the offspring after further adjusting for offspring antibiotics use during the first life year.

	**Diverse (n=8388)**	**Non-recommended (n=14110)**	**Mediterranean (n=14700)**	**Animal-based (n=3808)**	**Western (n=15091)**
Inflammatory bowel disease					
Unadjusted HR (95% CI)	0.58 (0.33-1.01)	0.97 (0.65-1.45)	0.81 (0.53-1.23)	0.72 (0.35-1.45)	Reference
Adjusted HR (95% CI)[1]	0.55 (0.31-0.97)	1.08 (0.70-1.66)	0.87 (0.56- 1.35)	0.76 (0.37-1.56)	Reference
Fully adjusted HR (95% CI)[2]	0.55 (0.31-0.97)	1.08 (0.70-1.67)	0.88 (0.57-1.37)	0.77 (0.38-1.57)	Reference
Crohn's disease					
Unadjusted HR (95% CI)	0.69 (0.32-1.48)	1.40 (0.82-2.38)	1.02 (0.57-1.80)	0.34 (0.08-1.43)	Reference
Adjusted HR (95% CI)[1]	0.67 (0.31-1.45)	1.41 (0.79-2.53)	0.98 (0.54-1.78)	0.34 (0.08-1.44)	Reference
Fully adjusted HR (95% CI)[2]	0.67 (0.31-1.47)	1.42 (0.79-2.54)	0.99 (0.55-1.81)	0.34 (0.08-1.45)	Reference
Ulcerative colitis					
Unadjusted HR (95% CI)	0.48 (0.21-1.10)	0.60 (0.32-1.13)	0.63 (0.34-1.17)	1.05 (0.46-2.42)	Reference
Adjusted HR (95% CI)[1]	0.44 (0.19-1.03)	0.73 (0.37-1.44)	0.77 (0.40-1.48)	1.20 (0.52-2.80)	Reference
Fully adjusted HR (95% CI)[2]	0.44 (0.19-1.03)	0.73(0.37-1.44)	0.77 (0.40-1.48)	1.21 (0.52-2.80)	Reference

*HRs refer to offspring's disease risk associated with the specific dietary pattern relative to the Western dietary pattern. [1]Adjusted for maternal educational level, pre-pregnancy body mass index, smoking during pregnancy, nutritional supplement use during pregnancy, energy intake during pregnancy, antibiotics use during pregnancy, and parental inflammatory bowel disease diagnosis. [2]Further adjusted for offspring's antibiotic use (yes/no) during the first life year.
